# Transcriptomic and epigenetic responses to short-term nutrient-exercise stress in humans

**DOI:** 10.1038/s41598-017-15420-7

**Published:** 2017-11-09

**Authors:** R. C. Laker, C. Garde, D. M. Camera, W. J. Smiles, J. R. Zierath, J. A. Hawley, R. Barrès

**Affiliations:** 1 Novo Nordisk Foundation Center for Basic Metabolic Research, University of Copenhagen, Copenhagen, Denmark; 2 Mary MacKillop Institute for Health Research, Centre for Exercise and Nutrition, Australian Catholic University, Melbourne, Australia; 3 Integrative Physiology, Department of Molecular Medicine and Surgery and Department of Physiology and Pharmacology, Karolinska Institutet, Stockholm, Sweden; 4 Research Institute for Sport and Exercise Sciences, Liverpool John Moores University, Liverpool, United Kingdom

## Abstract

High fat feeding impairs skeletal muscle metabolic flexibility and induces insulin resistance, whereas exercise training exerts positive effects on substrate handling and improves insulin sensitivity. To identify the genomic mechanisms by which exercise ameliorates some of the deleterious effects of high fat feeding, we investigated the transcriptional and epigenetic response of human skeletal muscle to 9 days of a high-fat diet (HFD) alone (Sed-HFD) or in combination with resistance exercise (Ex-HFD), using genome-wide profiling of gene expression and DNA methylation. HFD markedly induced expression of immune and inflammatory genes, which was not attenuated by Ex. Conversely, Ex markedly remodelled expression of genes associated with muscle growth and structure. We detected marked DNA methylation changes following HFD alone and in combination with Ex. Among the genes that showed a significant association between DNA methylation and gene expression changes were PYGM, which was epigenetically regulated in both groups, and ANGPTL4, which was regulated only following Ex. In conclusion, while short-term Ex did not prevent a HFD-induced inflammatory response, it provoked a genomic response that may protect skeletal muscle from atrophy. These epigenetic adaptations provide mechanistic insight into the gene-specific regulation of inflammatory and metabolic processes in human skeletal muscle.

## Introduction

Skeletal muscle function is critical for voluntary movement, heat production and energy homeostasis^1^. The role of skeletal muscle in metabolism and the control of blood glucose is particularly important, since this organ is responsible for up to 80% of whole body insulin-stimulated glucose uptake^2^. Skeletal muscle is also highly adaptive and displays a robust molecular and morphological response to diet and habitual physical activity^3,4^. High fat diets are detrimental for the function of metabolic tissues, including skeletal muscle. Indeed, increases in circulating lipids that accompany a fat-rich diet results in lipid accumulation within metabolic tissues, disruption to normal mitochondrial function, impaired insulin signalling and loss of muscle mass^5–7^. However, high-fat, low-carbohydrate diets have become popular regimes to achieve weight loss, mainly due to the satiating properties of these fatty acids. Resistance exercise promotes muscle hypertrophy and strength through the activation of signalling pathways that ultimately increase muscle protein synthesis^8^. Additionally, citrate synthase, hexokinase^9^ and muscle-specific lipid oxidation capacity^10^ is increased following resistance exercise in human skeletal muscle. Whether resistance exercise confers protection to skeletal muscle under conditions of a high-fat, low-carbohydrate diet has not been investigated. Early transcriptomic responses following the transition from a normal ‘healthy’ diet to a high fat diet (HFD) may provide important information as to the initial adaptive responses that result in loss of muscle mass and metabolic dysfunction. We therefore determined whether resistance exercise, in conjunction with high-fat feeding in humans, prevents maladaptive transcriptomic responses typically observed after such diets.

Altered DNA methylation has previously been linked to metabolic dysfunction in skeletal muscle of people with type 2 diabetes^11^ and in response to acute, intense exercise^12^. Therefore, to further probe the regulatory mechanisms responsible for skeletal muscle gene expression in response to diet/nutrient stimuli, we investigated the epigenetic modification of DNA methylation. We hypothesized that altered DNA methylation may be an epigenetic mechanism responsible for exercise and/or high-fat diet-induced adaptations in skeletal muscle. Indeed, changes in CpG methylation within promoters or enhancers can alter DNA structure and thereby block the access of transcriptional machinery to DNA, resulting in altered or suppressed gene expression.

We performed transcriptomic and genome-wide DNA methylation profiling in human skeletal muscle before and after nine days of HFD with or without three bouts of resistance exercise training in middle-aged, sedentary males. We report that diet- and exercise-induced changes in DNA methylation were associated with very specific gene regulation in the post-intervention resting state. Our findings also demonstrate the robust impact of resistance exercise on transcriptional remodelling in skeletal muscle, which may compensate for the deleterious effects of high-fat diets. This study provides insight into the possible initiating mechanisms of HFD-induced inflammation, metabolic dysfunction and loss of tissue mass in skeletal muscle.

## Research Design and Methods

### Study participants and experimental design

**Figure 1:**
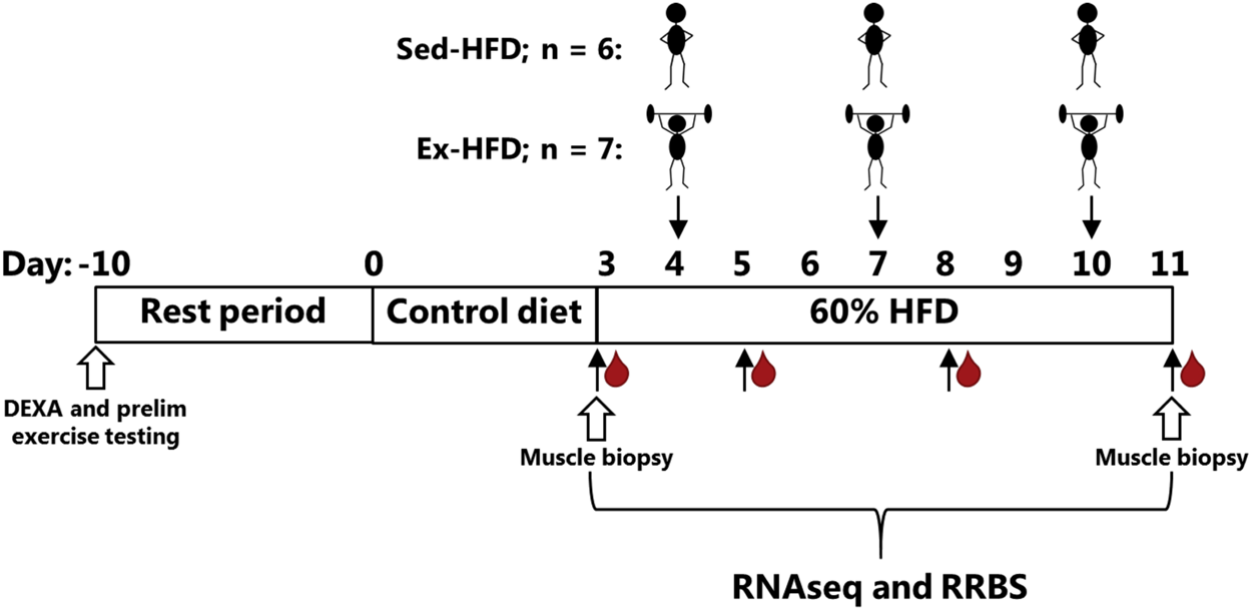
Study design. Subjects underwent body composition and exercise testing at the commencement of the experimental period. After 3 days of dietary control, subjects consumed a high-fat low-carbohydrate diet (HFD) for 9 days. Subjects either remained sedentary (Sed-HFD) or performed resistance exercise (Ex-HFD) on days 4, 7 and 10 of the experimental period. Blood samples were collected on days 3, 5, 8 and 11 and skeletal muscle biopsies were obtained before and after the diet/exercise intervention.

Thirteen healthy middle-aged sedentary men were recruited for this study. Body weight and BMI were in the normal range (Table 1). Participants were provided with oral and written information about the purpose, nature and potential risks involved with the study, and written informed consent was obtained prior to participation. All experimental protocols and methodologies related to the study were approved by the Australian Catholic University Human Research Ethics Committee (#2015-103 H, clinical trial registration date 12/10/2015) and conformed with the policy statement regarding the use of human subjects in the latest revision of the Declaration of Helsinki. The trial was registered with the Australian New Zealand Clinical Trials Registry (ACTRN 369316). A timeline of the experimental protocol that encompassed a parallel groups design is shown (Fig. 1). Ten days prior to the start of an intervention, all participants underwent DEXA scan (GE Lunar Prodigy Pro, GE Healthcare) to determine body composition, and preliminary exercise testing consisting of peak aerobic power (VO2peak) and one repetition maximum leg extension and leg press strength testing. At the commencement of the experimental period, all participants were provided with a standardized pre-packed control diet (breakfast, lunch, dinner and snacks) for three days. This diet was customized to the subject to provide 45 kcal/kg fat-free mass (FFM) per day with 6.1 g carbohydrate/kg FFM (55% total caloric intake), 1.7 g protein/kg FFM (15%) and 1.5 g fat/kg FFM (30%). Following an overnight fast, biopsies were collected under local anaesthesia (2-3 mL 1% Xylocaine) from the *vastus lateralis* muscle using a 5-mm Bergstrom needle, modified with suction, and denoted as the “Pre” time point. Participants then commenced a high-fat low-carbohydrate (HFD) diet consisting of 0.8 g carbohydrate kg/FFM (8% total caloric intake), 1.7 g protein/kg FFM (15%) and 3.9 g fat/kg FFM (77%)^13^ for the remaining experimental period, which was a further 9 days. This diet has been promoted by others to induce nutritional ketosis and to be beneficial for athletic performance and weight loss^13,14^. This diet contains the required minerals to maintain physiological function, protein for lean body mass and sufficient carbohydrates for brain function^13^. We found this diet had no impact on muscle protein turnover in the study participants and published the results elsewhere^15^. Meal plans were created using Foodworks 7.0 ® Xyris Software (Melbourne, Australia). Compliance was monitored and participants maintained a food checklist. Participants were divided into two groups that were pair matched for fat-free mass and strength: participants who remained sedentary (Sed-HFD) and those that performed three bouts of resistance exercise training (Ex-HFD), starting 1 day after commencing HFD, which corresponds to days 4, 7 and 10 of the experimental timeline (Fig. 1). Exercise consisted of 4 × 8–10 repetitions of leg press at 80% 1-RM, 4 × 8–10 repetitions of leg extensions at 80% 1-RM, and 4 sets of dumbbell squats. There was a 3 min recovery period between each set. Muscle biopsies were collected under fasted conditions on day 11 and denoted as the “Post” time point. Blood samples were collected on the morning of day 3, 5, 8 and 11 in EDTA tubes, centrifuged at 1,000 × g at 4 °C for 15 min and stored at −80 °C. Total RNA and DNA were simultaneously isolated from the biopsy using the Allprep® DNA/RNA/miRNA Universal Kit (Qiagen) according to the manufacturer’s instructions.

**Table 1:** Baseline characteristics of the participants.

	Ex-HFD (n = 7)	Sed-HFD(n = 6)
Age (y)	37.3 ± 5.6	38.8 ± 5.3
Body Mass (kg)	89.4 ± 12.8	84.5 ± 7.4
BMI (kg m^−2^)	26.9 ± 3.0	27.2 ± 2.5
Lean Mass (kg)	60.1 ± 6.0	55.7 ± 5.9
VO_2peak_ (ml/kg/min)	38.4 ± 4.2	34.5 ± 6.0
Leg Extension 1-RM (kg)	79.6 ± 15.4	71.8 ± 18.7
Leg Press 1-RM (kg)	219.7 ± 17.9	216.0 ± 49.2

Values are given as mean ± SD.

### Plasma Analysis

Plasma tumour necrosis factor α (TNF-α) and interleukin-6 (IL-6) were measured on 96-well plates utilizing commercially available and customised Milliplex Human magnetic bead panels (Millipore, Massachusetts, USA) following the kit-specific protocols provided by Millipore. Analytes were quantified in duplicate using the Magpix system utilising xPONENT 4.2 software. Concentrations of these analytes were determined on the basis of the fit of a standard curve for mean fluorescence intensity versus pg/ml. Two quality controls with designated ranges were run with each assay to ensure validity of data generated. Plasma FFA concentrations were determined by an enzymatic colorimetric method (Wako Diagnostics, Tokyo, Japan).

### RNA sequencing

RNA was checked for quality using the Agilent RNA 600 nano kit and Bioanalyser instrument (Agilent Technologies). 1 µg of RNA per sample was subject to the Illumina TruSeq Stranded Total RNA with Ribo-Zero Gold protocol (Illumina) and performed as described^16^. Briefly, ribosomal RNA was removed from the sample using 35 µl rRNA removal beads (Illumina) on a magnetic plate followed by clean-up of the ribosomal-depleted RNA with 193 µl Agencourt RNAClean XP beads (Beckman Coulter), 70% ethanol wash and elution into 10 µl Elution buffer (Illumina). The RNA sample was fragmented for 4 min at 94 °C in Elute, Prime, Fragment High Mix (Illumina) and then subject to first strand cDNA synthesis with 1 µl Superscript III reverse transcriptase (Life Technologies) per sample and thermocycler programmed to 25 °C for 10 min, 50 °C for 15 min and 70 °C for 15 min. Second strand cDNA was synthesized by addition of Second Strand Marking Master Mix and samples subject to 16 °C for 60 min. Samples were subject to another bead clean up prior to A-tailing and ligation of adapters as per kit instructions (Illumina). Following an third bead clean-up samples were enrich for DNA fragments by amplification using the Illumina PCR Primer Cocktail and PCR Master Mix using a pre-defined cycle number based on each individual sample and subject to 98 °C for 30 mins then X cycles of 98 °C for 10 secs, 60 °C for 30 secs and 72 °C for 30 secs and finally 72 °C for 5 min. Samples were cleaned and validated for DNA concentration using the Qubit dsDNA HS assay kit (Invitrogen) and for base pair size and purity using the Aglient High Sensitivity DNA chip and Bioanalyser instrument. Libraries were subjected to 100-bp single-end sequencing on the HiSeq 2500 (Illumina) at the Danish National High-Throughput DNA Sequencing Centre. Approximately 8.5 million reads/sample were assigned to genes with 23,373 genes surviving the expression threshold.

### DNA methylation analysis

Reduced Representation Bisulfite Sequencing (RRBS) was performed as described^17^. Briefly, 200 ng of DNA per sample was incubated overnight at 37 °C with MpsI enzyme (NEB #R0106L) to fragment DNA at CCGG positions to enrich for CpG regions. Samples then underwent gap filling and A-tailing with 1 µl dNTP mix (10 mM dATP, 1 mM dCTP, 1 mM dGTP) and 1 µl Klenow fragment 3′−5′ exo (NEB) with 30 °C for 20 min and 37 °C for 20 min. Samples underwent bead clean-up using 90 µl AMPure beads (Beckman Coulter) on a magnetic plate, 2 × 70% ethanol wash and elution into 20 µl elution buffer. Illumina Truseq adapters (diluted 1:20) were ligated with T4 ligase (NEB) and overnight incubation at 16 °C. The enzyme was deactived by incubation at 65 °C for 20 mins. Samples were pooled (12 samples per pool), volume adjusted with 20% polyethylene glycol and 2.5 M NaCl prior to bead clean-up in the DynaMag magnet. Bisulfite conversion was performed using the EZ DNA methylation Kit (Zymo Research) according to the manufacturer’s instructions with 20 hr incubation with CT conversion reagent at 50 °C. DNA was then PCR amplified using Pfu Turbo hotstart DNA polymerase, dNTP mix (100 mM, 25 mM each) and Illumina primer cocktail. Samples were subject to 2 min at 95 °C followed by 14 cycles of 95 °C for 30 sec, 65 °C for 30 sec and 72 °C for 45 sec and finally 72 °C for 5 min. Samples underwent final bead clean-up and library validation for DNA concentration using the Qubit dsDNA HS assay kit (Invitrogen) and base pair size and purity using the Aglient High Sensitivity DNA chip and Bioanalyser instrument. Libraries were subjected to 100-bp single-end sequencing on the HiSeq 2500 (Illumina) at the Danish National High-Throughput DNA Sequencing Centre.

### Accession Numbers

Sequencing data are archived for public access at the Gene Expression Omnibus (http://www.ncbi.nlm.nih.gov/geo) under accession number GSE99965.

### Bioinformatic analysis

RNA-seq reads were subjected to trimming of adapters and low quality flanking ends using Trim Galore v0.3.7 and Cutadapt v1.4.2. Pre-processed reads were mapped to hg38 using Rsubread^18^ and gene coverages were computed with featureCounts^19^ and the Gencode annotation. The gene list was filtered to those with a read coverage larger than 0.1 rpkm in at least 5 samples. Differential expression was computed using edgeR with the glmQLFit/glmQLFTest modeling framework and the following models y~Timepoint + Subject were used for each of the HFD-Ex and HFD-Sed groups^20^. Genes with a false discovery rate (FDR) below 0.1 were considered differentially expressed.

RRBS reads were processed with the ‘rrbs’ setting of Trim Galore v0.3.7 and Cutadapt v1.4.2. Processed reads were mapped to hg38 followed by derivation of CpG methylation using Bismark^21^. Differentially methylated regions (FDR < 0.1) were identified using BiSeq^22^ from the subset of CpG sites that are covered by at least half of the samples using the following models y~Timepoint + Subject for each of the HFD-Ex and HFD-Sed groups. Our confidence in bisulfite conversion efficiency was assessed based on the level of non-CpG methylation, which at CHG sites averaged 1.41% and at CHH sites averaged 1.39%, which is in the expected range (Fig. S1). We also identified CpG sites known to be highly or lowly methylated in adult human skeletal muscle as well as 50 other tissue and cell types using RRBS data available from the Epigenome Roadmap. We found that the methylation levels of these sites within our analysis were consistent with the expected levels (Fig. S2). Finally, we assessed the distribution of methylation of identified CpG sites for each sample, which was bimodal with peaks in the 0–15% and 85–100% range (Fig. S3).

### Statistics

Enrichment studies were conducted using hypergeometric tests and corrected for multiple testing using the Benjamini-Hochberg procedure. An FDR of <0.1 was used as significance level. Two-way ANOVA followed by Student Newman Kuel’s post-hoc tests were performed to determine differences between Ex-HFD and Sed-HFD groups, and time (Pre-and Post). Data are presented as mean ± SEM with P values < 0.05 indicating statistical significance.

## Results and Discussion

### Short-term resistance exercise causes major changes in the skeletal muscle transcriptome compared with HFD alone

**Figure 2:**
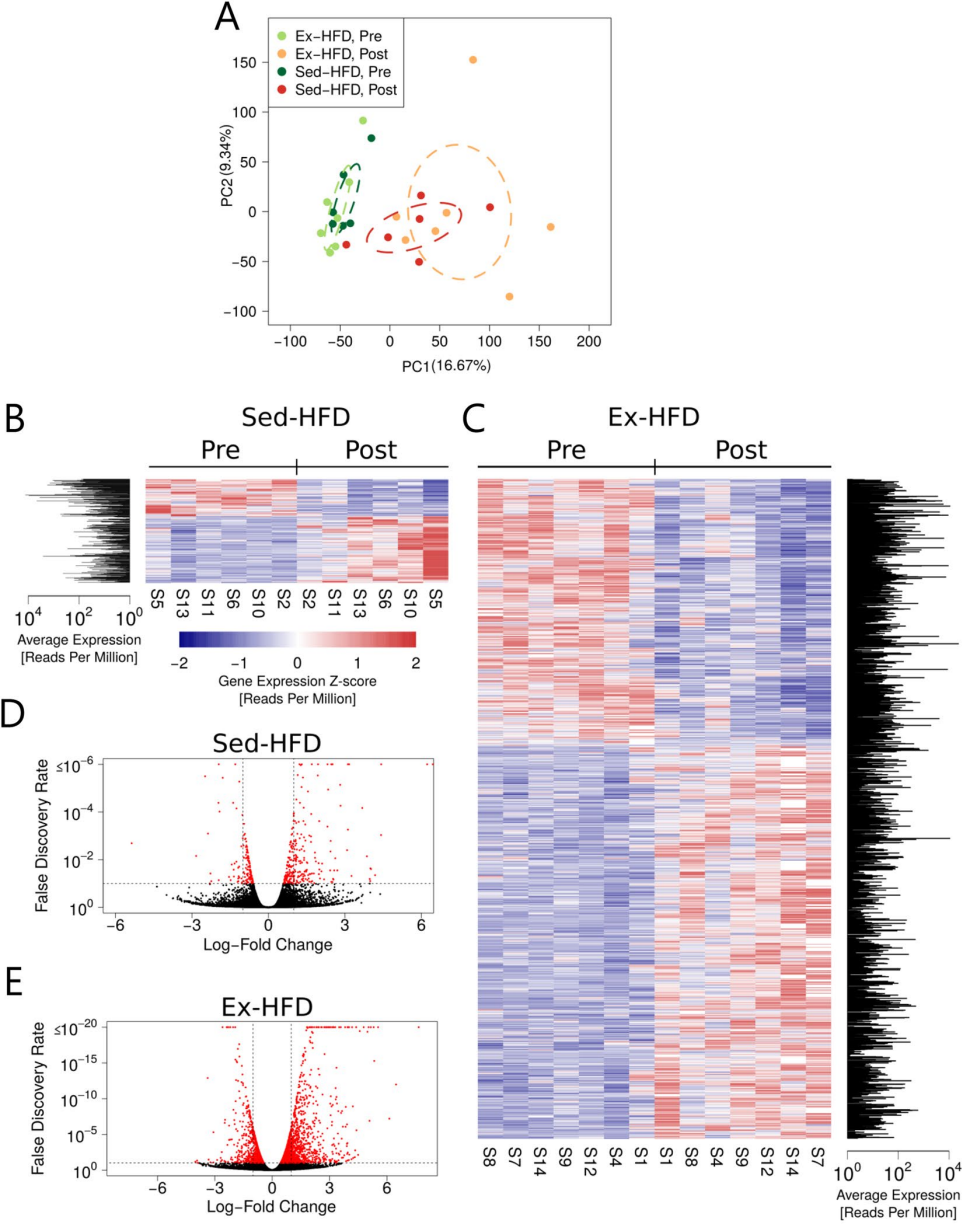
Short-term resistance exercise initiates robust transcriptional regulation compared with HFD alone. (**A**) Principal component analysis (PCA) of RNA-seq for the major two principal components (PC). The 95% confidence ellipses are shown for each group. Heatmaps (**B** and **C**) and volcano plots (**D** and **E**) represent differentially expressed genes in skeletal muscle before (Pre) and after (Post) 9 days of HFD (Sed-HFD; **B** and **D**) or HFD with 3 bouts of resistance exercise training (Ex-HFD; C and E). FDR < 0.1.

To identify regulatory mechanisms involved in the early adaptive response to high-fat feeding and the interaction with resistance exercise, we profiled the skeletal muscle transcriptome before and after 9 days of HFD, with or without 3 bouts of resistance exercise. Within the RNA-seq analysis, 23,373 genes were annotated. Principal component analysis showed a clear separation of the treatment groups between the Pre and Post time points, with no clear separation between individuals that performed resistance exercise and individuals that remained sedentary after the intervention (Fig. 2A). To confirm the accuracy of our RNA-seq results we compared the expression profile of genes previously analysed by qRT-PCR in a subset of samples from the same experiment, which were previously reported^15^. We found that both the differentially expressed and unchanged genes were consistent between the two analyses (Fig. S4). Nine days of HFD in sedentary men (Sed-HFD) resulted in differential expression of 412 genes in skeletal muscle (Pre *vs*. Post), with 264 up-regulated and 148 down-regulated (Fig. 2B and D; Table S1). Conversely, when resistance exercise was performed in combination with HFD (Ex-HFD), a greater transcriptomic response was evident, with 2,617 genes changed (Pre *vs*. Post) of which 1,561 were up-regulated and 1,056 were down-regulated (Fig. 2C and E; Table S1). The different magnitude of change in gene expression suggests that resistance exercise initiates robust transcriptional activity in skeletal muscle, which far outweighs the impact of HFD alone.

**Figure 3:**
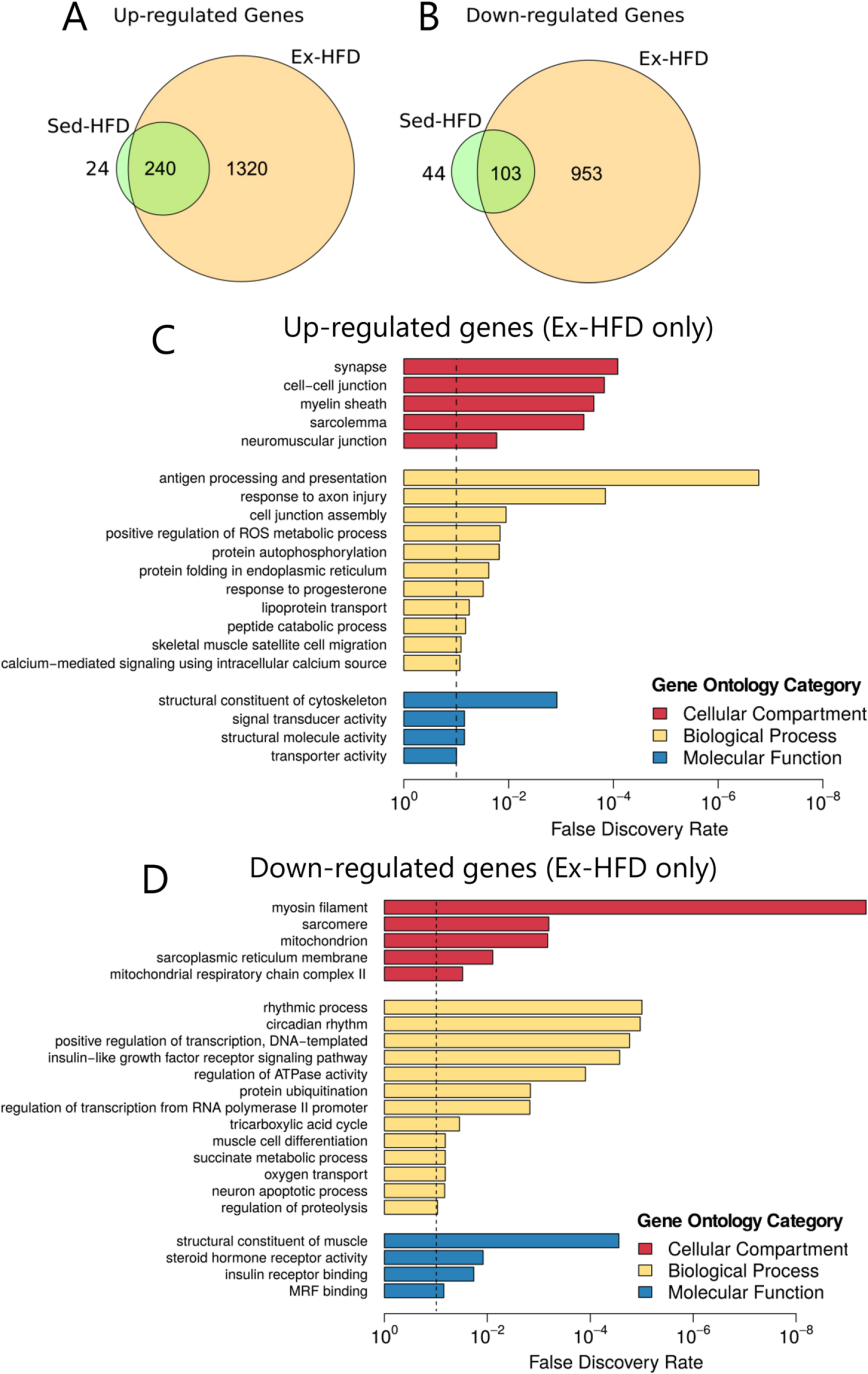
Short-term resistance exercise is associated with large-scale transcriptional remodelling in skeletal muscle in the presence of HFD. Venn diagrams representing the number of genes that were up-regulated (**A**) and down-regulated (**B**) in Sed-HFD and Ex-HFD group (excludes CHAC1 gene, which was upregulated in Ex-HFD and downregulated in Sed-HFD). The intersection represents the genes that were regulated following both interventions. Gene ontology analysis of genes that were up-regulated (**C**) and down-regulated (**D**) exclusively in the Ex-HFD group. FDR < 0.1 is shown by the dotted line.

Of the 1,561 up-regulated genes following Ex-HFD, only 240 genes were also up-regulated in the Sed-HFD group. This implies that the remaining 1,321 genes were up-regulated as a direct response to resistance exercise (Fig. 3A). Similarly, of the 1,056 genes down-regulated in the Ex-HFD group, only 103 genes were also down-regulated in the Sed-HFD group, suggesting that resistance exercise was primarily responsible for the down-regulation of the remaining 953 genes. We performed an enrichment analysis of gene ontology (GO) terms to identify whether the genes regulated in the Ex-HFD group were associated with specific cellular compartments, biological processes and molecular functions (Table S2). However, due to the large number of differentially expressed genes, we retrieved a long list of GO terms. Therefore, we used the Revigo tool to summarize the GO terms based on semantic similarity^23^. We found many GO terms related to skeletal muscle structure, myogenic activity and metabolism (Fig. 3C and D; Table S2). Consistent with the biology of skeletal muscle, we found that the cellular compartment GOs were related to *neuromuscular junction*, *sarcomere* and *mitochondrion* (Fig. 3C and D; Table S2), while biological process GOs included *skeletal muscle satellite cell migration*, *cell junction assembly* and *muscle cell differentiation*, among other metabolic and transcriptional processes (Fig. 3C and D; Table S2). Finally, molecular function gene ontologies were related to *signal transducer activity*, *myogenic regulatory factor binding* and *structural constituent of muscle* (Fig. 3C and D; Table S2). Of note, the top two GOs of the down-regulated genes were *rhythmic process* and *circadian rhythm* (Fig. 3D; Table S2). Since the muscle biopsies were taken at the same time of the day (~8am), pre- and post-intervention, this suggests that resistance exercise may transcriptionally regulate the innate circadian oscillations of skeletal muscle. This may be important considering the close link between circadian and metabolic gene regulation^24,25^ and could have widespread implications for the timing of exercise to optimize metabolic health outcomes. Taken collectively, our analyses suggest that resistance exercise may protect skeletal muscle against the negative impact of HFD. However, we acknowledge that functional outcomes are difficult to determine based on the short-term exercise intervention in the current study. Of note, metabolic analyses from this cohort revealed the Ex-HFD group showed a tendency for improved glucose tolerance^15^. The functional impact of resistance exercise, when performed in conjunction with HFD, may become more apparent after a prolonged exercise training regimen.

### Short-term HFD induces immune and inflammatory genes regardless of physical activity

**Figure 4:**
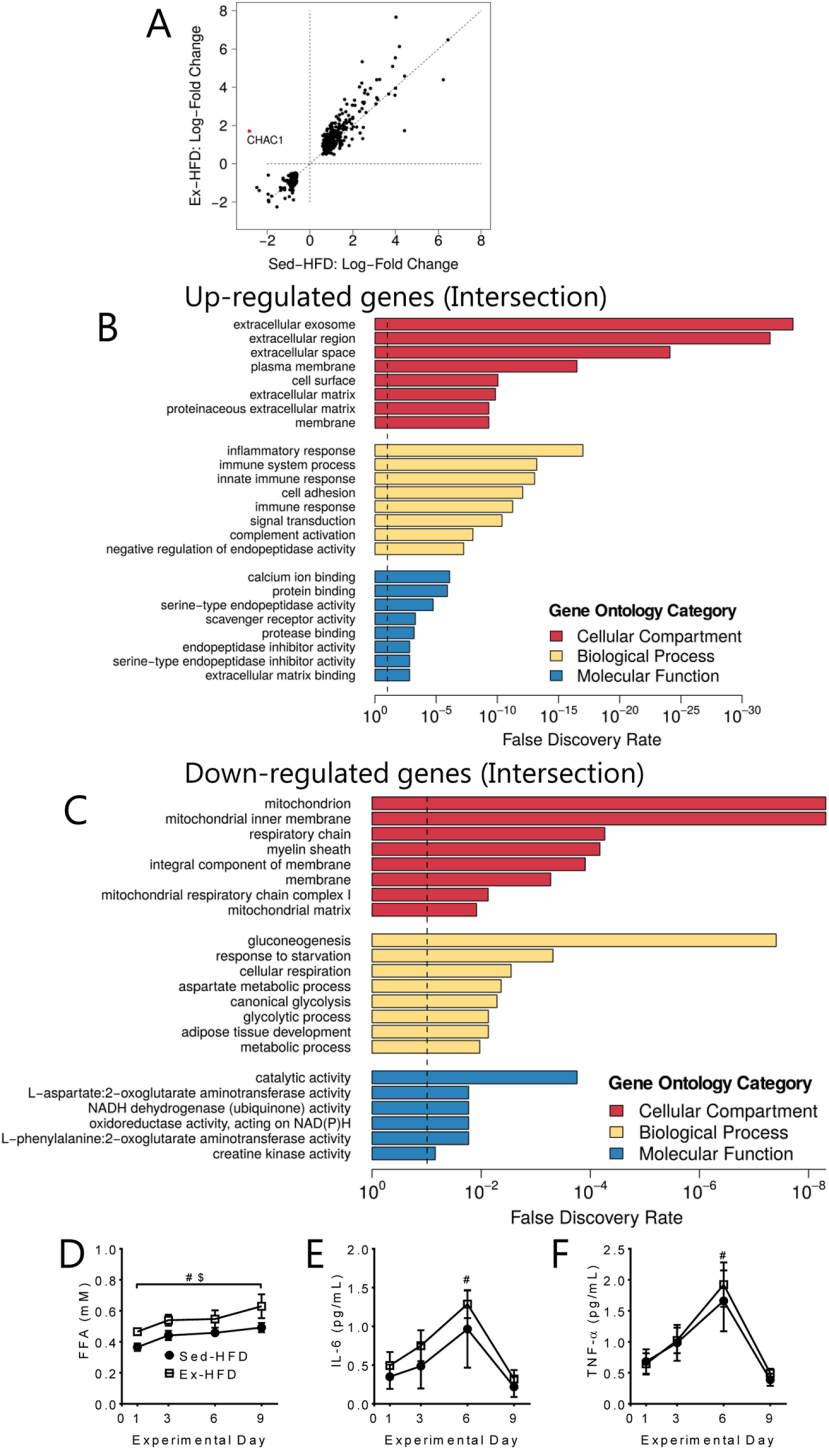
HFD induces immune and inflammatory genes associated with systemic inflammation, regardless of physical activity. (**A**) Scatter plot of 344 differentially expressed gene following 9 days of HFD, with or without resistance exercise (Sed-HFD and Ex-HFD intersection). Gene ontology analysis of the 344 genes that were either up-regulated (**B**) or down-regulated (**C**) in both the Sed-HFD and Ex-HFD groups. FDR < 0.1 is shown by the dotted line. Plasma profiles of free fatty acids (FFA; **D**), interleukin 6 (IL-6; **E**) and TNF-α (**F**) throughout the intervention period. ^#^*p < *0.002 for the effect of time; ^$^*p* < 0.02 for the effect of exercise.

We identified 343 genes that were differentially regulated following both interventions (Sed-HFD and Ex-HFD; Fig. 4A), with 240 genes up-regulated and 103 genes down-regulated (Fig. 3A and B). Only one gene, ChaC Glutathione Specific Gamma-Glutamylcyclotransferase 1 (CHAC1), showed divergent transcriptional regulation between Sed-HFD and Ex-HFD groups (Fig. 4A). CHAC1 plays a role in glutathione degradation, notch signalling and activation of autophagy and apoptosis^26–28^. To our knowledge, the only report on CHAC1 in skeletal muscle suggests that CHAC1 is induced in response to re-feeding and participates in the unfolded protein response^29^, which is consistent with our observation that *CHAC1* is regulated by the nutritional state in humans. Next, we performed gene ontology analysis of the 344 genes differentially regulated by both Sed-HFD and Ex-HFD. We identified at least 20 individual GO terms associated with immune and inflammatory processes (Fig. 4B and C). Of potential interest, many of the up-regulated GO terms were associated with the extracellular space, which suggests that immune and inflammatory signalling is occurring outside the skeletal muscle, likely through a combination of secreted and membrane-expressed proteins, as well as recruitment of macrophages. Whether macrophage recruitment or muscle damage *per se* is driving the immune and inflammatory gene response after a combined HFD and resistance exercise regimen warrants further investigation. Within the cellular compartment category, the down-regulated GO terms were highly associated with mitochondria, and supports the notion that HFD induces impairments in mitochondrial function (Fig. 4C). Indeed, many of the biological function GOs terms derived from the down-regulated genes were associated with mitochondrial function and metabolic processes including *cellular respiration*, *metabolic process*, *gluconeogenesis*, *glycolytic process* and *canonical glycolysis* (Fig. 4C).

In support of the transcriptional profiling data, we found that circulating free fatty acids progressively increased throughout the high-fat diet intervention in both groups (Fig. 4D). Surprisingly, this occurred to a greater extent in the exercise group compared with the sedentary group (Fig. 4D). We also observed a marked elevation of the inflammatory markers IL-6 and TNF-α in both groups (Fig. 4E and F). Collectively, these findings suggest that HFD, regardless of physical activity, elevates circulating lipids associated with systemic inflammation, and promotes local inflammation/immune responses in skeletal muscle. These observations suggest that concomitant resistance exercise does not fully protect skeletal muscle from deleterious HFD-induced gene regulation. We cannot exclude the possibility that a longer exposure to an exercise stimulus (i.e. weeks, months) may reverse this response.

### Transcriptional response to short-term HFD

We report that 67 genes were uniquely altered in the Sed-HFD group and unchanged in the Ex-HFD group. This number excludes the CHAC1 gene which was altered in both groups but in opposite directions (see Fig. 4A). This finding suggests that resistance exercise preserved expression of these genes at a basal level. Although we were unable to perform gene ontology analysis due to the small number of genes, we found that many of these 67 genes were associated with mitochondrial function and localization, metabolic enzymes, and immune regulation (Table 2). Of particular interest are the Interleukin 1 Receptor Associated Kinase 2 (IRAK2), Nuclear Protein 1, Transcriptional Regulator (NUPR1; also known as p8) and Fibroblast Growth Factor 6 (FGF6) (Table 2). IRAK2 was increased by ~2-fold following HFD. IRAK2 is a receptor for IL1 and mediates toll like receptor (TLR) and NF-ĸB signalling to induce transcription and mRNA stabilization for chemokine production (TNFα, IL6, IL1)^30,31^. The early induction of *IRAK2* by HFD feeding suggests that IRAK2 participates in the initiation of inflammatory signalling in skeletal muscle^32–34^ and may be a key mechanism underlying subsequent insulin resistance. *NUPR1* was also induced following HFD by ~1.7-fold. NUPR1 interacts directly with the critical myogenic regulatory factor MyoD to regulate MyoD target genes^35^. In C2C12 myoblasts, over expression of NUPR1 represses MyoD and myogenin gene expression^35^. Furthermore, NUPR1 is involved in resistance to stress induced by a change in the microenvironment^36,37^. Thus, our observation that *NUPR1* is induced following HFD may represent a stress response to the influx of fatty acids, which ultimately results in the HFD-induced loss of muscle mass. Finally, FGF6 was decreased ~50% following nine days of HFD in skeletal muscle. FGF6 plays an role in muscle regeneration and differentiation through stimulation of satellite cell proliferation and early differentiation^38–40^. We speculate that the decrease of *FGF6* along with increased *NUPR1* participates in the loss of muscle mass with HFD.

**Table 2:** Selected genes associated with mitochondrial function and localization, metabolic enzymes, and immune regulation differentially regulated by HFD and preserved by concomitant resistance exercise.

Ensembl ID	Gene	Description	logFC	P value	FDR
ENSG00000124107	SLPI	secretory leukocyte peptidase inhibitor	2,82	7,17E-09	7,28E-06
ENSG00000138193	PLCE1	phospholipase C epsilon 1	0,94	2,22E-05	4,80E-03
ENSG00000014641	MDH1	malate dehydrogenase 1	−0,79	3,68E-05	7,17E-03
ENSG00000134070	IRAK2	interleukin 1 receptor associated kinase 2	1,01	5,31E-05	8,94E-03
ENSG00000176046	NUPR1	nuclear protein 1, transcriptional regulator	0,76	8,99E-05	1,29E-02
ENSG00000154518	ATP5G3	ATP synthase, H + transporting, mitochondrial Fo complex subunit C3 (subunit 9)	−0,74	1,07E-04	1,46E-02
ENSG00000112715	VEGFA	vascular endothelial growth factor A	−0,73	1,47E-04	1,88E-02
ENSG00000087586	AURKA	aurora kinase A	−1,14	1,56E-04	1,95E-02
ENSG00000110955	ATP5B	ATP synthase, H + transporting, mitochondrial F1 complex, beta polypeptide	−0,71	2,88E-04	3,13E-02
ENSG00000159423	ALDH4A1	aldehyde dehydrogenase 4 family member A1	−0,68	5,48E-04	4,75E-02
ENSG00000263232	ATP5A1P3	ATP synthase, H + transporting, mitochondrial F1 complex, alpha subunit 1 pseudogene 3	−0,71	6,70E-04	5,32E-02
ENSG00000132313	MRPL35	mitochondrial ribosomal protein L35	−0,70	7,28E-04	5,63E-02
ENSG00000111241	FGF6	fibroblast growth factor 6	−1,05	8,14E-04	6,18E-02
ENSG00000169692	AGPAT2	1-acylglycerol-3-phosphate O-acyltransferase 2	0,73	8,36E-04	6,18E-02
ENSG00000176340	COX8 A	cytochrome c oxidase subunit VIIIA (ubiquitous)	−0,63	9,09E-04	6,48E-02
ENSG00000166343	MSS51	MSS51 mitochondrial translational activator	−0,71	1,02E-03	7,01E-02
ENSG00000184076	UQCR10	ubiquinol-cytochrome c reductase, complex III subunit X	−0,61	1,23E-03	7,98E-02
ENSG00000244482	LILRA6	leukocyte immunoglobulin-like receptor, subfamily A (with TM domain), member 6	2,51	1,43E-03	8,74E-02
ENSG00000260318	COX6CP1	cytochrome c oxidase subunit VIc pseudogene 1	−1,05	1,72E-03	9,85E-02
ENSG00000120992	LYPLA1	lysophospholipase I	−0,90	1,72E-03	9,85E-02

In addition to the set of known genes that were differentially expressed following HFD, there was a set of eight transcripts with no identified protein product (Table 3). These transcripts exhibited an extremely robust decrease in expression following HFD (between ~40–98% reduction; Table 3). The transcripts are quite long and could therefore, be long non-coding RNA that could confer a transcriptional response. Alternatively, some of these genes appear to be subject to splicing and may be host genes to microRNAs, which are transcribed, spliced and then degraded. In any case, the function of these transcripts remains unknown and they may play a critical role in maintaining mass and/or metabolic homeostasis in skeletal muscle. An important future direction will be to determine if these HFD-regulated genes also participate in HFD-induced inflammation, metabolic dysregulation or loss of muscle mass, and how resistance exercise prevents this HFD-induced genetic regulation.

**Table 3:** Un-annotated genes down-regulated by HFD and protected by concomitant resistance exercise.

Ensembl ID	Gene	logFC	P value	FDR
ENSG00000261303	RP11–160C18.2	−5,36	6,96E-06	2,06E-03
ENSG00000262420	RP11–490O6.2	−1,44	1,19E-04	1,60E-02
ENSG00000224550	RP11–270C12.3	−0,72	2,21E-04	2,58E-02
ENSG00000272631	RP11–359E3.4	−2,27	3,88E-04	3,81E-02
ENSG00000271347	RP11–701H24.7	−1,48	1,07E-03	7,27E-02
ENSG00000277954	RP11–679B19.1	−1,18	1,22E-03	7,98E-02
ENSG00000223935	AC008074.3	−2,24	1,26E-03	8,00E-02
ENSG00000183154	RP11–863K10.7	−2,30	1,74E-03	9,91E-02

### Epigenetic response to HFD and resistance exercise

**Figure 5:**
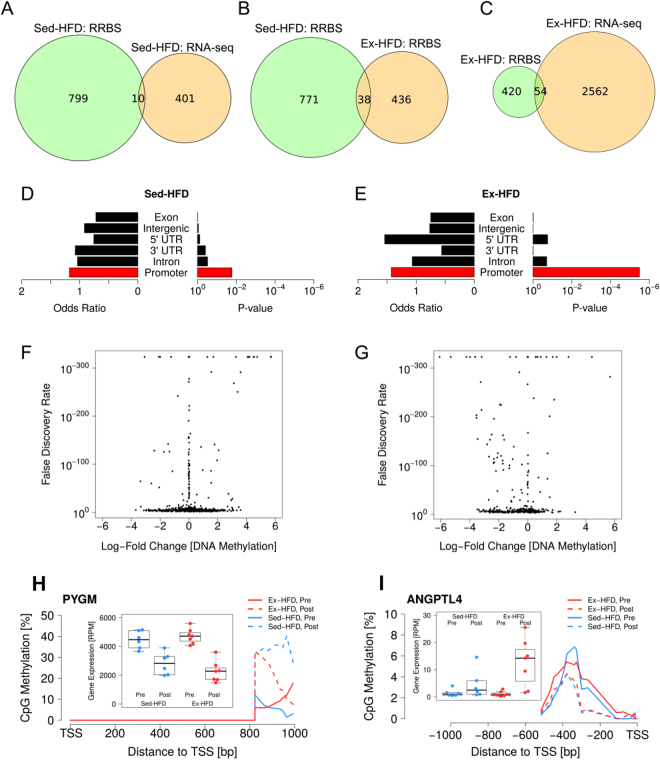
Differential DNA methylation following short-term HFD with or without resistance exercise. Venn diagrams representing the number of genes that were differentially methylated and differentially expressed following 9 days of HFD (**A**) and with concomitant resistance exercise (**C**). Venn diagram showing the number of differentially methylated genes between Sed-HFD and Ex-HFD groups (**B**). Genomic context of DMRs detected in the Sed-HFD (**D**) and Ex-HFD (**E**) groups. Volcano plots showing directional changes and FDR of DMRs in the Sed-HFD (**F**) and Ex-HFD (**G**) groups. DNA methylation level relative to transcription start site (TSS) and gene expression (insert) for the genes PYGM (**H**) and ANGPTL4 (**I**).

We used RRBS to profile differentially methylated DNA regions (DMRs) with the aim of investigating whether epigenetic mechanisms play a role in the transcriptomic response to nutrient/exercise. We found that a HFD alone induced a greater degree of hypermethylation, while concomitant resistance exercise resulted in a preference towards hypomethylation of DNA (Fig. 5F and G). We found 809 DMRs following short-term HFD, while concomitant resistance exercise revealed 474 DMRs, with only 38 DMRs common between the two groups (Fig. 5A–C). Gene ontology analysis of all DMRs revealed only one term, which was *regulation of transcription*. Furthermore, we identified only 10 genes in the Sed-HFD group and 54 genes in the Ex-HFD group that showed significant association with differential gene expression (Fig. 5A and C). This limited relationship is surprising considering that more DMRs were found in promoters, compared with other genomic regions (Fig. 5D and E), and theoretically would cause altered expression of the genes associated with these promoters. One could speculate that the specific location of the DMR within the promoter region will be an important factor dictating whether gene expression would be altered, which is dependent on the recruitment of methy-CpG binding proteins specifically to the transcription factor binding sites to block their access. Another explanation is that the DMRs have no apparent impact unless there is a stimulus to initiate transcriptional activity of the associated gene, following which the functional impact of DNA methylation or demethylation can be realized. Indeed, in the present study, biopsies were taken 48 hours after the final exercise bout, when skeletal muscle was in a resting/basal state with low levels of transcriptional activity. Therefore, the differential methylation states identified may have regulatory functions for transcriptional activation/repression during times of stress or stimulation. For example, DNA demethylation following resistance exercise training may function to poise a promoter region for rapid transcriptional activation in response to a subsequent exercise bout, as previously hypothesized^41^. We propose that the importance of DNA methylation for exercise adaptation may be observed if the biopsy was sampled during a dynamic period of transcription such as immediately after exercise.

The glycogen phosphorylase, muscle associated (PYGM) gene was one of the genes that did show a relationship between expression and promoter methylation. The promoter of PYGM was hypermethylated in both groups following HFD, regardless of exercise and the level of methylation was inversely correlated with gene expression (Fig. 5H). PYGM is an enzyme involved in the breakdown of glycogen to glucose-1-phosphate^42,43^. The epigenetic regulation of PYGM may be an adaptive response to the high-fat low-carbohydrate diet, since glycogen stores are expected to progressively decline in skeletal muscle and the requirement for an enzyme that breaks down glycogen (i.e. PYGM) would be abolished in the absence of the substrate. Indeed, the decline in muscle glycogen stores has been reported in human muscle following a similar diet for 4 weeks^14^. Meanwhile, angiopoiten like 4 (ANGPTL4) was hypomethylated in both groups, with changes in gene expression only observed following resistance exercise, but to a substantial degree (Fig. 5I). ANGPTL4 is a secreted serum hormone that regulates blood glucose, lipid metabolism and insulin sensitivity^44–46^. Hypomethylation of ANGPTL4 may be a compensatory response to HFD, yet a stimulus such as exercise may be required for transcriptional activation. Interestingly, ANGPTL4 induces lipolysis in adipocytes^44.^ Thus, increased expression of *ANGPTL4* in the Ex-HFD group may contribute to the higher levels of FFAs as compared with the sedentary group provided with the same HFD.

In conclusion, we have reported transcriptional profiles in skeletal muscle from men fed a high-fat diet with or without a concomitant resistance exercise intervention. The extent to which resistance exercise can prevent the deleterious impact of HFD on skeletal muscle function remain unanswered, but the dramatic number of exercise-responsive genes identified suggest that growth and development pathways are up-regulated, along with changes in metabolism and transcription. We found significant changes in DNA methylation, predominantly at promoter regions. Furthermore, genes associated with the DMRs were generally unrelated between the Sed-HFD and Ex-HFD groups and their functional significance has yet to be determined. Overall, our findings suggest that resistance exercise may be a promising intervention to maintain skeletal muscle mass and metabolic health under conditions of a high-fat diet, currently prevalent throughout the world. Future studies are warranted to investigate the long-term functional outcomes of HFD and resistance exercise combinations, as well as dynamic exercise studies to dissect the epigenome-transcriptome relationship in skeletal muscle.

## Electronic supplementary material

Supplemental file

Supplementary tables

Supplementary tables
