# Author Correction: Transcriptomic and epigenetic responses to short-term nutrient-exercise stress in humans

**DOI:** 10.1038/s41598-018-23227-3

**Published:** 2018-03-19

**Authors:** R. C. Laker, C. Garde, D. M. Camera, W. J. Smiles, J. R. Zierath, J. A. Hawley, R. Barrès

**Affiliations:** 1 Novo Nordisk Foundation Center for Basic Metabolic Research, University of Copenhagen, Copenhagen, Denmark; 2 Mary MacKillop Institute for Health Research, Centre for Exercise and Nutrition, Australian Catholic University, Melbourne, Australia; 3 Integrative Physiology, Department of Molecular Medicine and Surgery and Department of Physiology and Pharmacology, Karolinska Institutet, Stockholm, Sweden; 4 Research Institute for Sport and Exercise Sciences, Liverpool John Moores University, Liverpool, United Kingdom

Correction to: *Scientific Reports* 10.1038/s41598-017-15420-7, published online 09 November 2017

The Acknowledgements section in this Article is incomplete.

“The Novo Nordisk Foundation Centre for Basic Metabolic Research is an independent research centre at the University of Copenhagen partially funded by an unrestricted donation from the Novo Nordisk Foundation.”

should read:

“This work was supported by a Novo Nordisk Foundation Challenge grant NNF14OC0011493. The Novo Nordisk Foundation Centre for Basic Metabolic Research is an independent research centre at the University of Copenhagen partially funded by an unrestricted donation from the Novo Nordisk Foundation.”

