# Supplementary material for: Transcriptomic and epigenetic responses to short-term nutrient-exercise stress in humans
Source: Sci Rep. 2017 Nov 9;7:15134. doi: 10.1038/s41598-017-15420-7 (PMC5680174; doi:10.1038/s41598-017-15420-7)
Supplement: Supplementary file 1 — Supplemental file [file 41598_2017_15420_MOESM1_ESM.pdf]

Supplementary information

Transcriptomic and epigenetic responses to short-term nutrient-exercise stress in humans

Laker, R.C.[1], Garde, C.[1], Camera, D.M[2], Smiles, W.J[2], Zierath, J.R.[1,3], Hawley, J.A.[2,4] and Barrès, R[1,*]

[1]Novo Nordisk Foundation Center for Basic Metabolic Research, University of Copenhagen, Denmark; [2]Mary MacKillop Institute for Health Research, Centre for Exercise and Nutrition, Australian Catholic University, Melbourne, Australia; [3]Integrative Physiology, Department of Molecular Medicine and Surgery and Department of Physiology and Pharmacology, Karolinska Institutet, Stockholm, Sweden; [4]Research Institute for Sport and Exercise Sciences, Liverpool John Moores University, Liverpool, United Kingdom

[*]Corresponding author: Romain Barrès, University of Copenhagen, Blegdamsvej 3B, 2200 Copenhagen, Denmark, Phone +45 35 33 71 10, barres@sund.ku.dk

Online Supplemental Material

Table S1. Differentially expressed genes and DMRs. Tab 1: RNA-seq Ex-HFD (FDR<0.1). Tab 2: RNA-seq Sed-HFD (FDR<0.1). Tab 3: RRBS Ex-HFD (FDR<0.1). Tab 4: RRBS Sed-HFD (FDR<0.1).

Table S2. RNA-seq GO analyses. Tab 1: Biological process upregulated genes. Tab 2: Biological process downregulated genes. Tab 3: Molecular function upregulated genes. Tab 4: Molecular function downregulated genes. Tab 5: Cellular compartment upregulated genes. Tab 6: Cellular compartment downregulated genes.

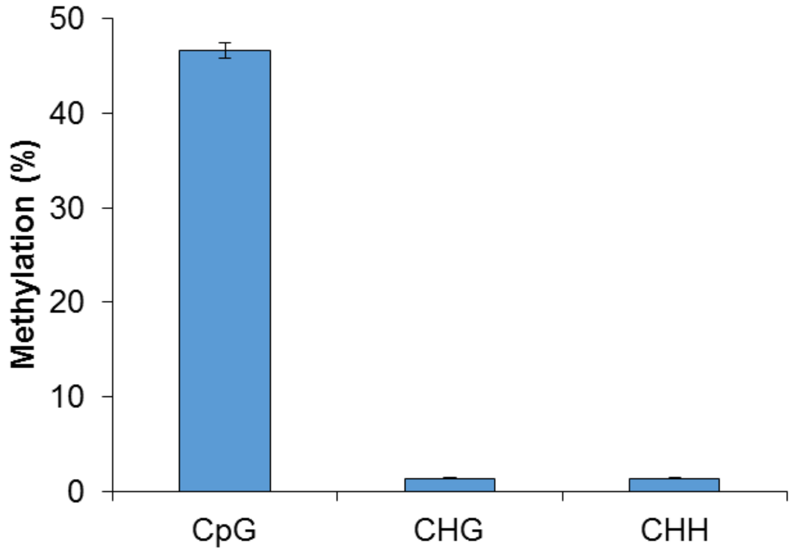

Figure S1. Percent methylation of CpG, CHG and CHH sites within our RRBS analysis. Non-CpG methylation of CHG and CHH sites averaged 1.41% and 1.39%, respectively.

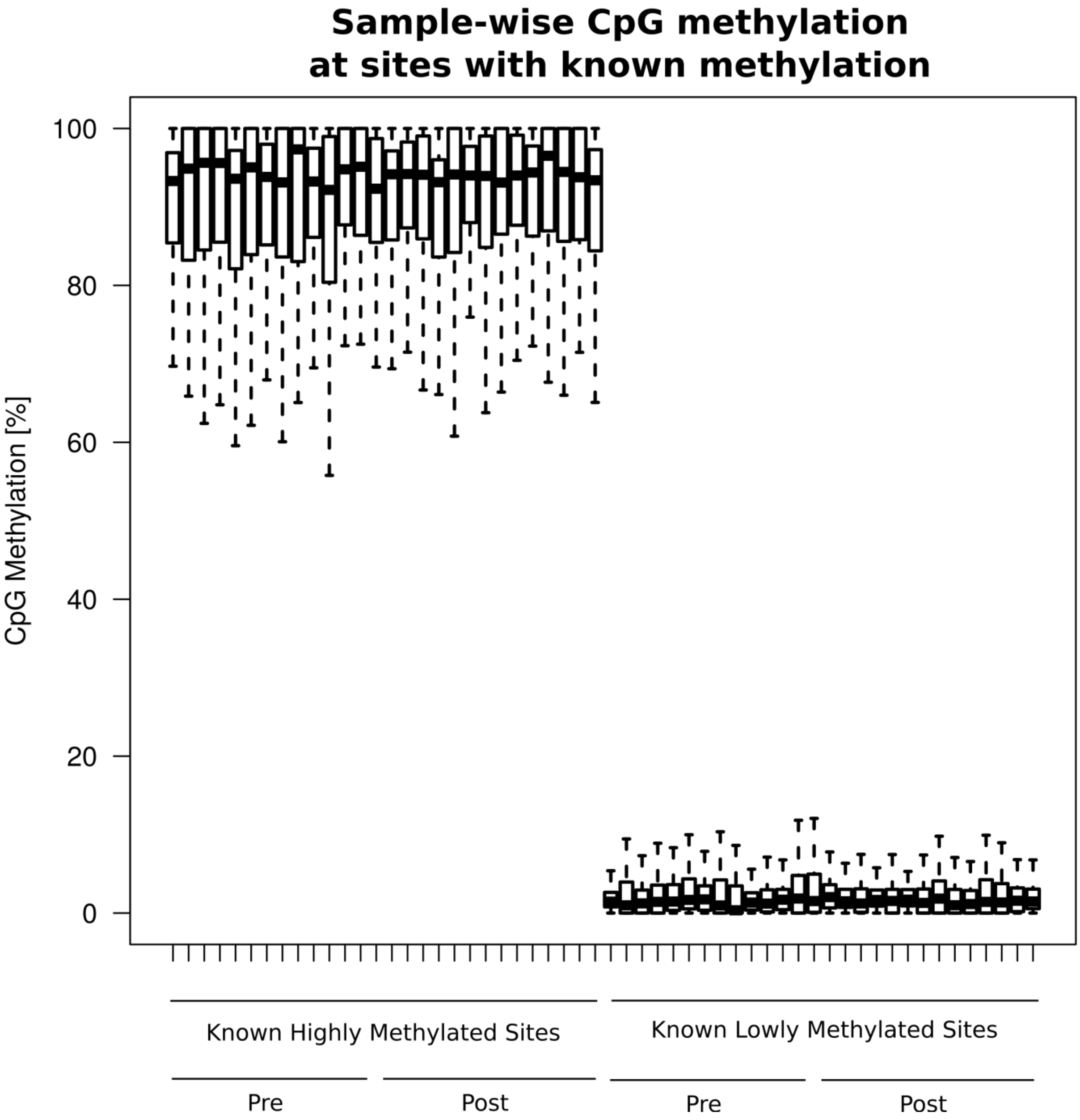

Figure S2. Percent methylation of CpG sites known to have either very high or very low methylation state in adult human skeletal muslce as well as across 50 different cell types. For each sample in our analysis, the methylation level was consistent with the expected methylation level for eath known CpG site.

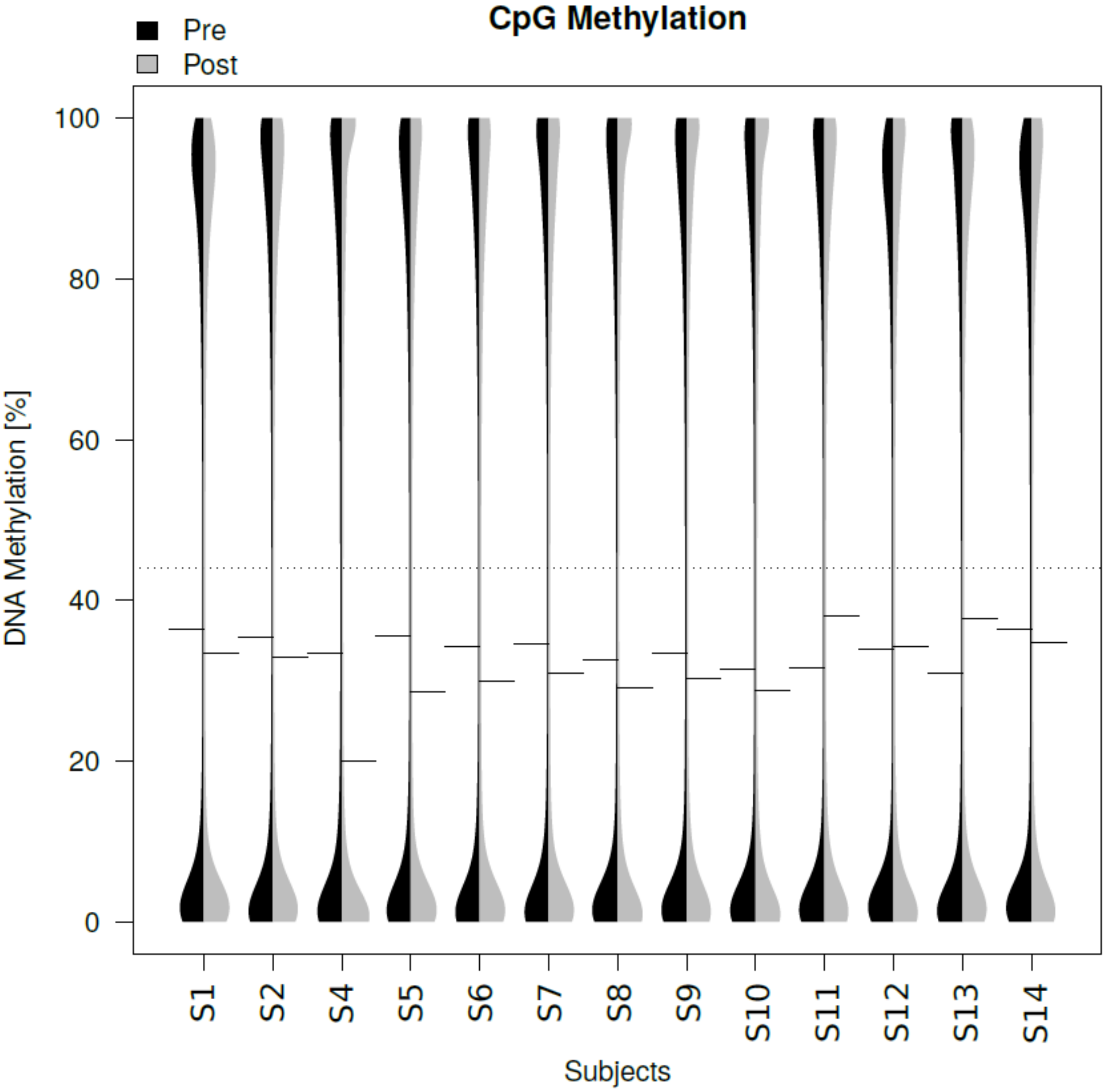

Figure S3. Distribution of % methylation of all CpG sites explored in our differential analysis for each participant. The CpG methylation had a bimodal distribution with the majority of CpG sites between 0-15% and 85-100% methylated, suggestive of successful bisulfite conversion. Average level of methylation for each sample is represented by the horizontal line.

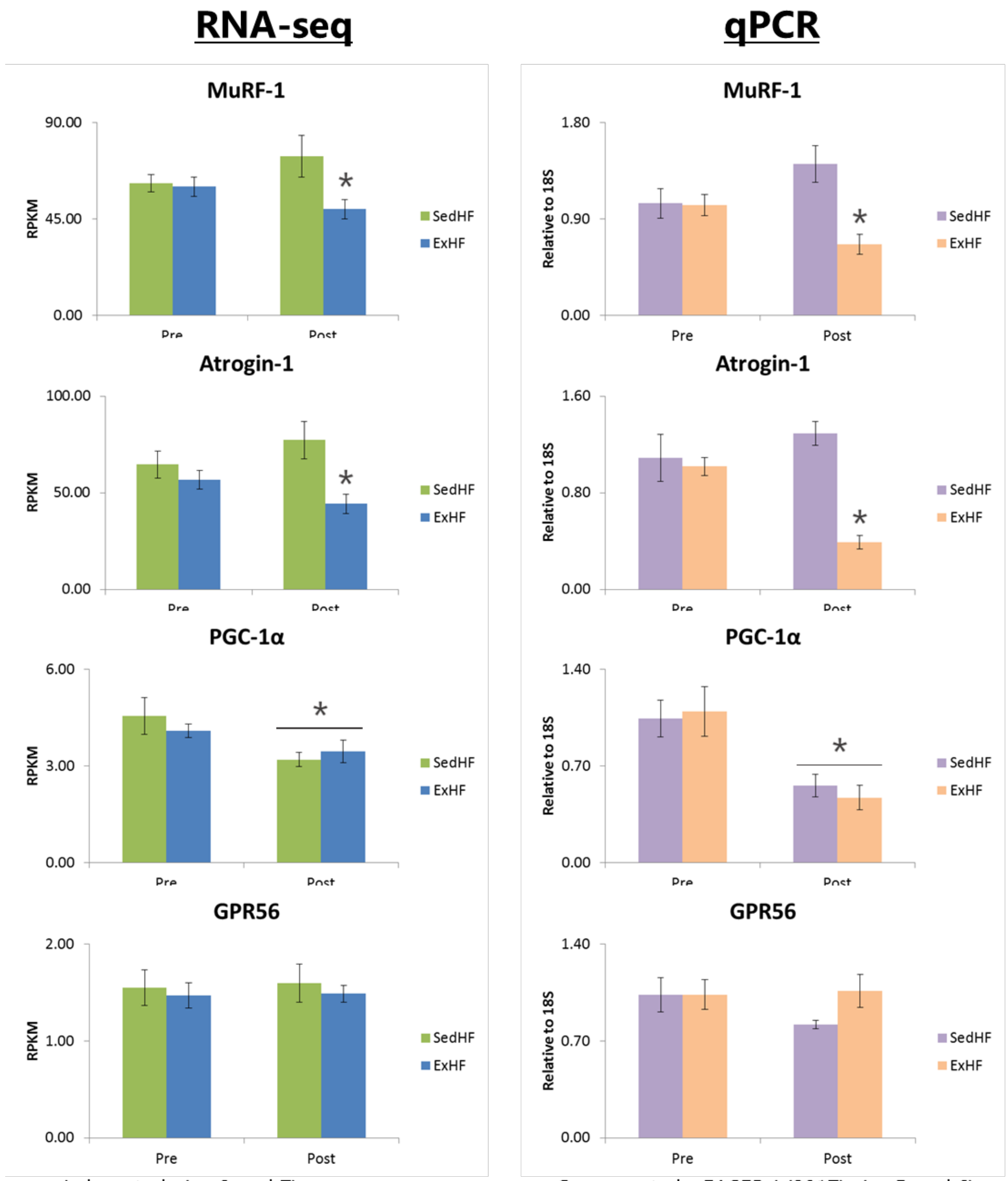

Laker et al., (n=6 and 7) Camera et al., *FASEB J*, (2017); (n=5 and 6)

Figure S4. Validation of RNAseq data using real-time qPCR. Raw data in reads per kilobase million (RPKM) was extracted from the RNAseq (Left) for selected genes, which had been previously analysed by real-time qPCR (right). The qPCR analysis was performed for a subset of samples from the same experiment (outlined in Fig. 1) and published elsewhere. We compared the expression patterns between the 2 analyses and found them to be consistent. The genes analyzed included (A) MuRF-1, (B) Atrogin-1, (C) PGC-1α and (D) GPR56.
